# Analysis of Epidemiological Characteristics of Scarlet Fever in Zhejiang Province, China, 2004–2018

**DOI:** 10.3390/ijerph16183454

**Published:** 2019-09-17

**Authors:** Qinbao Lu, Haocheng Wu, Zheyuan Ding, Chen Wu, Junfen Lin

**Affiliations:** Department of Public Health Surveillance & Advisory, Zhejiang Provincial Center for Disease Control and Prevention, 3399 Binsheng Road, Binjiang District, Hangzhou 310051, China; qblu@cdc.zj.cn (Q.L.); hchwu@cdc.zj.cn (H.W.); zhyding@cdc.zj.cn (Z.D.); chenwu@cdc.zj.cn (C.W.)

**Keywords:** scarlet fever, epidemic characteristics, spatiotemporal analysis, Zhejiang Province

## Abstract

Objective: The aim of this study was to analyze the trends and epidemiological characteristics of scarlet fever in Zhejiang Province in 2004–2018, intending to provide a basis for targeted prevention and control of this disease. Method: We collated the epidemiological data for cases of scarlet fever from the China Information System for Disease Control and Prevention (CISDCP) in Zhejiang province between 1 January 2004 and 31 December 2018. Descriptive statistical analysis was used to analyze epidemiological characteristics of scarlet fever, whereas the Getis-Ord Gi* statistic was used to determine the hotspot incidence of scarlet fever. Results: In 2004–2018, a total of 22,194 cases of scarlet fever were reported in Zhejiang Province, with no death reports. The annual average of scarlet fever incidence was 2.82/100,000 (range,1.12 to 6.34/100,000). The male incidence was higher than that among female (χ^2^ = 999.834, *p* < 0.05), and a majority of the cases (86.42%) occurred in children aged 3–9 years. Each year, the incidence of scarlet fever in Zhejiang Province appeared two seasonal peaks: the first peak occurred from March to June (the constituent ratio was 49.06%), the second peak was lower than the first one during November and the following January (the constituent ratio was 28.67%). The two peaks were almost in accordance with the school spring semester and autumn–winter semester, respectively. The incidence in the northern regions of the province was generally higher than that in the southern regions. High-value clusters were detected in the central and northern regions, while low-value clusters occurred in the southern regions via the Getis-Ord Gi* statistical analysis. Conclusions: The prevalence of scarlet fever in Zhejiang Province showed a marked seasonality variation and mainly clustered in the central and northern regions in 2004–2018. Children under 15 years of age were most susceptible to scarlet fever. Kindergartens and primary schools should be the focus of prevention and control, and targeted strategies and measures should be taken to reduce the incidence.

## 1. Introduction

Scarlet fever is an acute respiratory infection caused by group A Beta hemolytic streptococcus, which usually occurs in winter and spring and commonly affects children [1]. Scarlet fever typically spreads in crowds through respiratory droplets or direct contact with the mucus, saliva, or skin of the infected persons. However, some outbreaks of scarlet fever are even found to be food-borne [2]. It is more prevalent in children aged 3–9 years [3], so clustered outbreaks are more common in overcrowded places, such as kindergartens and schools. It is worth noting that currently no vaccine is available to prevent scarlet fever [4]. With antibiotic treatment, scarlet fever can be completely cured, and complications can be prevented.

In recent years, scarlet fever has begun to once again prevail in some countries due to factors such as changes in the virulence of group A streptococcus. In the past decade, many countries and regions such as Australia, the United Kingdom, Poland, South Korea, Hong Kong, and Taiwan have reported scarlet fever epidemics [5,6,7,8,9,10]. The incidence of scarlet fever in the Chinese mainland has also shown a sharp upward trend [11].

Scarlet fever is one of the Class B infectious diseases that must be reported according to the Law of the People’s Republic of China on the Prevention and Control of Infectious Diseases [12]. It is also an infectious disease with high incidence and key prevention and control requirements in China. In 2006, about 1000 cases of scarlet fever outbroke in a primary school in Ningbo, Zhejiang Province [2]. In 2011, a large-scale scarlet fever epidemic occurred in Hong Kong [13], with more than 900 cases of scarlet fever and 6 cases of toxic shock syndrome (including 2 deaths). The situation of prevention and control is challenging.

The incidence of scarlet fever in Zhejiang Province has increased since 2004, especially since 2011. In this paper, we presented a descriptive analysis and Getis-Ord Gi* statistical analysis of the spatiotemporal epidemiology of scarlet fever in Zhejiang Province. The results might indicate the transmission trends and confirm the high-risk regions of scarlet fever, providing reliable information for future surveillance and control efforts of scarlet fever in Zhejiang Province. The results are reported as follows.

## 2. Materials and Methods

### 2.1. Data Source

The China Information System for Disease Control and Prevention (CISDCP) was initiated in 2004, which is the most comprehensive and macroscopic notifiable disease surveillance system in China [12]. Notifiable infectious disease cases have been reported to this system in real time [14].

The data of scarlet fever cases were extracted all the clinical and laboratory diagnosed cases of scarlet fever from the CISDCP, with the symptom onset date between 1 January 2004 and 31 December 2018 and the current living addresses of the subjects in Zhejiang Province. The diagnosis of scarlet fever is based on the diagnostic criteria issued by the Ministry of Health of the People’s Republic of China (WS 282-2008) [15]. The population data of all ages were obtained from the subsystem “Basic Information System” of the CISDCP, which is updated annually.

### 2.2. Study Area

Zhejiang Province, located on the southeast coast of the China, consists of 11 cities, and has moist air, a mild climate, and a developed economy. According to the geographical orientation and landform features, we divided the 11 cities into 5 parts. The central and northern parts of Zhejiang Province are plain areas (the central region: Hangzhou, Shaoxing, and Jinhua; the northern region: Jiaxing and Huzhou), the Southern and Western parts are hilly and mountainous areas (the southern region: Wenzhou, Lishui, and Taizhou; the western region: Quzhou), and the eastern parts are coastal areas (the western region region: Ningbo and Zhoushan). It covers an area of 101,800 km^2^ and is one of the most densely populated provinces in China. By 2018, the population has reached up to 56 million, and the population aged 0–14 years was about 7.6 million.

### 2.3. Statistical Methods

The scarlet fever cases were distributed in 11 cities in Zhejiang Province. The descriptive analysis method was used to analyze the incidence, number of cases, and composition ratio of scarlet fever in Zhejiang Province in 2004–2018. The reported incidence rate (R) of scarlet fever cases was calculated by dividing the number of reported scarlet fever cases (C) via the CISDCP by the number of inhabitants (I) registered in local public health facilities (R = C/I). The reported incidence rates were calculated per 100,000 persons. The incidence rates by male or female sex presented as per 100,000 of that sex category. Chi-square test was used to compare count data. A *p* < 0.05 was considered statistically significant.

The Getis-Ord Gi* statistic was used to determine the hotspot incidence of scarlet fever. The Getis-Ord Gi* statistic belongs to the category of local spatial autocorrelation analysis methods [16,17], which indicated high-value clusters (hot spots) and low-value clusters (cold spots) of scarlet fever in the entire study location. The Gi* statistic can be used as a measure of the degree of spatial clustering. The degree of clustering and its statistical significance is estimated based on a confidence level, according to the Z-scores and *p*-values. They indicate whether the observed spatial clustering of high or low values is different from the random distribution. If the Z (Gi*) score of the unit area is high and positive with *p* < 0.05, it indicates a hotspot (a high-value spatial cluster) in this region. On the contrary, if the Z (Gi*) score is low and negative with *p* < 0.05, it indicates a cold-spot (a low-value spatial cluster). The Z (Gi*) score is higher, the clustering degree is greater. If the Z (Gi*) score is close to zero, it indicates no obvious spatial clustering. Regions with Z-scores > 2.58 are considered to be significant at 99% confidence level (*p* < 0.01), and they are classified as the highest risk regions. The Z-scores between 1.65–1.96 and 1.96–2.58 are considered to be significant at 90% and 95% confidence levels (*p* < 0.10, and 0.05), and they are considered as high-risk regions (hot spots). Whereas, Z-scores < −1.65 means low values clusters (cold spots) [18].

All analyses were performed using Statistical Package for the Social Sciences, version 16.0 (SPSS, Chicago, IL, USA), ArcGIS software (version 10.1, ESRI Inc., Redlands, CA, USA) and Excel 2016 (Microsoft, Redmond, WA, USA).

### 2.4. Ethical Statement

This study was exempt from institutional review board assessment. Scarlet fever is one of the Class B notifiable infectious diseases. Firstly, policy documents and statistics data related to notifiable disease on public websites of China Health Department, CISDCP, Zhejiang Health Department, and Zhejiang Statistical Bureau were collected and understood. Secondly, data were acquired from secondary sources and analyzed anonymously, therefore no participant was required to provide written informed consent.

## 3. Results

### 3.1. General Characteristics of Scarlet Fever

A total of 22,194 cases of scarlet fever were reported in Zhejiang Province during the period of 2004–2018, with no deaths and an average annual incidence rate of 2.82/100,000. There were almost clinically diagnosed cases which accounted for 99.31% (22,040/22,194).

### 3.2. Temporal Distribution

**Figure 1:**
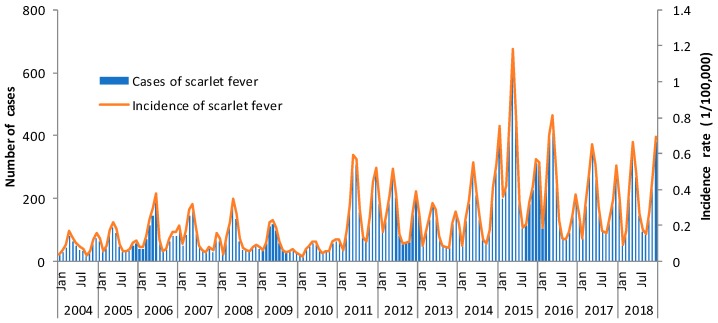
Monthly incidence and reported cases of scarlet fever in Zhejiang Province in 2004–2018.

In 2004–2018, the reported incidence rate of scarlet fever showed an upward long-term trend (χ^2^_trend_ = 4082.561, *p* < 0.05) in Zhejiang Province, from 1.12/100,000 in 2004 to 4.08/100,000 in 2018, and the highest annual incidence rate (6.34/100,000) was in 2015 (Figure 1). The incidences of scarlet fever were in a relatively stable state fluctuating from 0.88 to 1.90/100,000 during 2004–2010. The incidence reached 3.57/100,000 in 2011, and thereafter remained 2.22 to 6.34/100,000 (2011–2018). There were reported cases of scarlet fever in Zhejiang Province every month. Each year, the incidence rate of scarlet fever appeared two seasonal peaks. The first peak occurred from March, and gradually reached the summit during April and June. A total of 10,888 cases were reported, accounting for 49.06% (10,888/22,194). The incidence gradually decreased and reached the trough in August. The second peak occurred during November and the following January, with a summit slightly lower than the first one. A total of 6363 cases were reported, accounting for 28.67% (6363/22,194). Then the trough following it appeared in February (Figure 1). The incidence of scarlet fever decreased during the school holidays in winter (February) and summer (August).

### 3.3. Population Distribution

**Figure 2:**
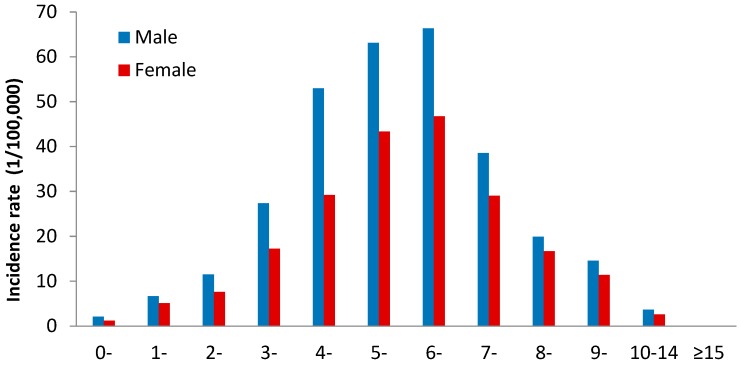
Age and sex distribution of morbidity of scarlet fever in Zhejiang Province, 2004–2018.

**Figure 3:**
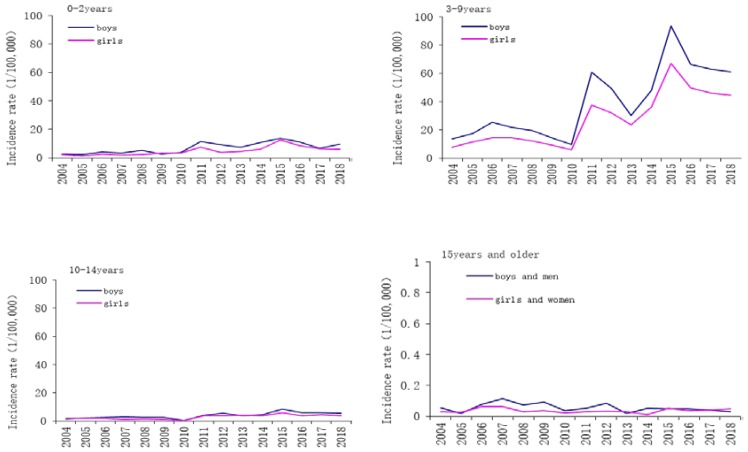
The incidence of scarlet fever in Zhejiang Province, by patient age group and sex. The scale for those aged 15 years and older was from 0~1 because the average incidence is so low.

In 2004–2018, a total of 13,715 male cases and 8479 female cases of scarlet fever were reported, with a male to female ratio of 1.62:1 (Table 1). Overall, we found that the incidence rates gradually decreased with the increasing age (χ^2^_trend_ = 130,082.8, *p* < 0.05), and adult incidence rate of was very low (Figure 2). The average annual male incidence was 3.39/100,000, and the average annual female incidence was 2.20/100,000. The incidence of male was higher than that of female (χ^2^ = 999.834, *p* < 0.05). In terms of occupational distribution, the cases were mainly kindergarten children (43.09%, 9563), followed by students (35.57%, 7894). In terms of age of onset, most scarlet fever cases were 3–9 years old (86.42%, 19,179) during the study period (Table 1). The annual incidence among patients aged 0–2 years peaked in 2011 and 2015, and the annual incidence among those aged 10–14 years peaked in 2012 and 2015, whereas the incidence among those aged 3–9 years peaked at three timepoints, in 2006, 2011, and 2015 (Figure 3). On the contrary, the annual incidence among cases aged ≥15 years remained substantially lower than the other age groups (Figure 3).

### 3.4. Spatial Distribution

**Figure 4:**
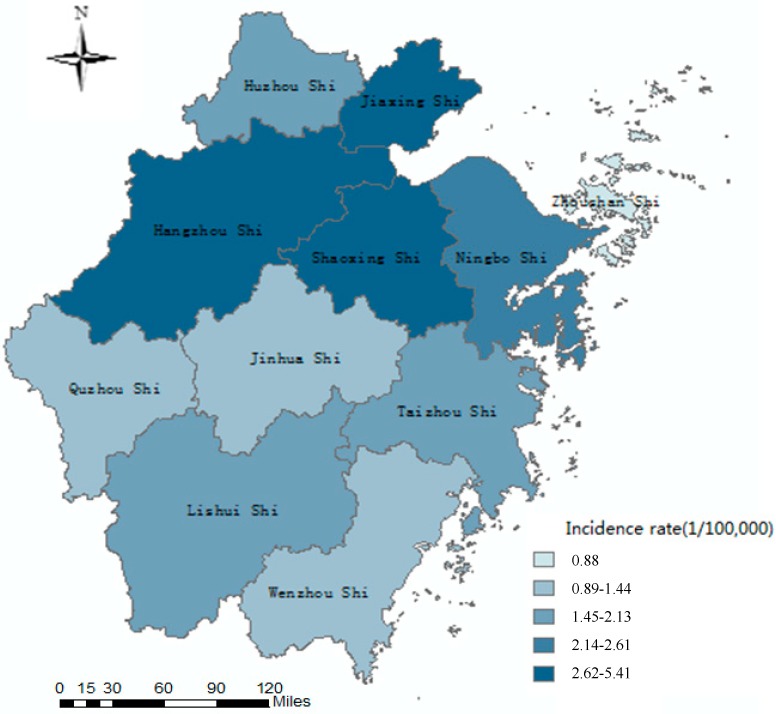
Geographic distribution of annual average incidence rate of scarlet fever in Zhejiang Province, 2004–2018.

In 2004–2018, scarlet fever cases were reported in 11 cities throughout the whole province, with the highest reported incidence in Shaoxing (5.41/100,000), Hangzhou (5.39/100,000), Jiaxing (4.38/100,000), Ningbo (2.61/100,000), and Huzhou (2.12/100,000). The top five cities reported cases were in Hangzhou (6341 cases), Shaoxing (3703 cases), Ningbo (2687 cases), Jiaxing (2637 cases), and Taizhou (1843 cases) (Figure 4).

**Figure 5:**
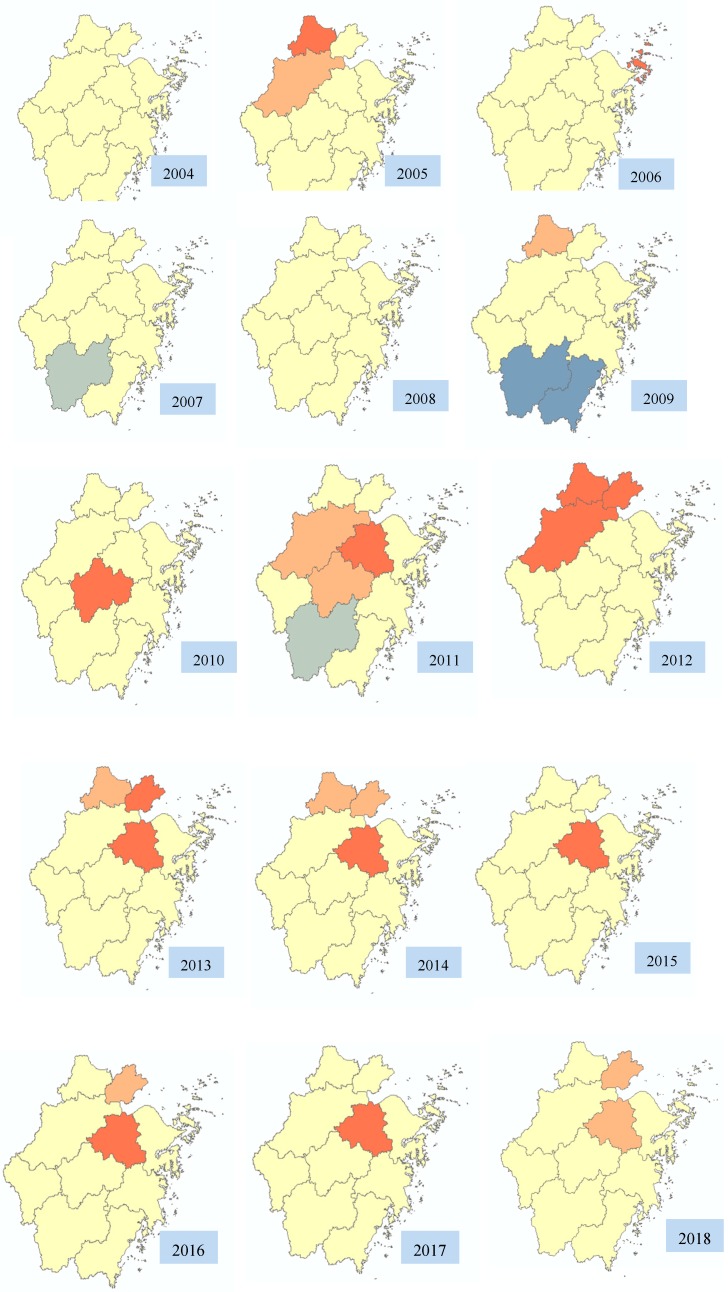

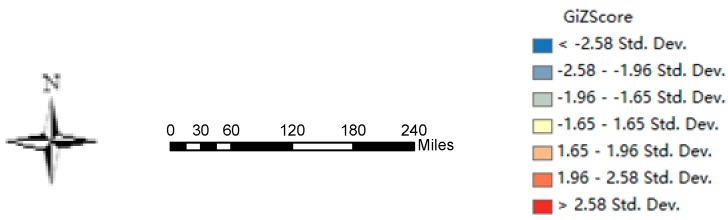
Clusters of scarlet fever incidence in Zhejiang Province, 2004–2018.

Figure 5 demonstrated the clusters of scarlet fever incidence at the district level in Zhejiang Province, 2004–2018. The location and size of high value clusters varied in each year. Generally, the epidemic characteristics of scarlet fever showed the incidence in the northern regions was generally higher than that in the southern regions. The high value clusters were detected in the central and northern regions, while low value clusters occurring in the southern regions via the Getis-Ord Gi* statistical analysis. During the study period, a total of 27 clustering areas were detected by Getis-Ord Gi* analysis, including 23 high-value clustering (hot spots) areas and 4 low-value clustering (cold spots) areas. High-value clustering areas were detected in the northern regions of Zhejiang Province (Huzhou and Jiaxing) in 7 years, respectively in 2005, 2009, 2012, 2013, 2014, 2016, and 2018. Since 2010, high-value clusters have been increasing in central Zhejiang (Hangzhou, Shaoxing, Jinhua). Before 2010, only one high-value cluster was detected in Hangzhou in 2005. While 11 clusters were detected during 2010–2018, mostly in Shaoxing (7 clusters), followed by Hangzhou (2 clusters) and Jinhua (2 clusters), which suggested a trend of the scarlet fever high value areas in Zhejiang Province extending from the northern to the central regions. In addition, only one high-value clustering was detected in Zhoushan in 2006, located on the eastern coast of Zhejiang Province, but no similar situation has occurred since then. Compared with the central and northern regions, the southern regions (Wenzhou, Lishui, Taizhou) had low incidence rates. There were four low-value clusters detected in 2007, 2009, and 2011, respectively, mostly in Lishui (three clusters). In addition, there was no high value clusters detected in southern regions in the past 15 years (Figure 5). Table 2 showed the location, Z-score, and *p*-value of clusters in Zhejiang Province (2004–2018). There was no signifcant area detected in 2004 and 2008, whereas high value clusters and low value clusters were detected in other years.

## 4. Discussion

Our research provides a good basis for understanding the epidemiological characteristics of scarlet fever in Zhejiang Province in 2004–2018. The surveillance results showed that the reported incidence rate of scarlet fever varied greatly with time in Zhejiang Province in the past 15 years. Overall, the incidence rate of scarlet fever showed an upward long-term trend from 2004 to 2018, and the highest annual incidence rate was in 2015. In the past 10 years, there were some studies suggesting that scarlet fever was showing a rapid upward trend in other countries [19,20,21]. This study showed that the trend was consistent with the other regions of China [3,11,18]. Some research findings indicated that meteorological factors, as well as air pollutant factors may increase the incidence of scarlet fever [22,23,24,25], such as nitrogen oxide, rainfall, and sunshine hour were significantly associated with scarlet fever incidence. However, the underlying mechanism remained unclear; further studies should be done to explore environmental factors.

This study also showed there were two epidemic peaks each year during the current study period. The two peaks were almost in accordance with the school spring semester (from March to June) and autumn–winter semester (from November to the following January), respectively. This was also similar to the study findings in Guangdong Province, China [26], suggesting that schools may have played an important role in the spread of scarlet fever. Combined with previous findings that school-age children were the major group with scarlet fever, we can conclude that kindergartens and primary schools should be the focus of surveillance and control of this infectious disease. It is well known that scarlet fever occurs mainly in winter/spring [27,28]. Some researchers believe that seasonal fluctuations of scarlet fever may be attributed to climatic conditions [23,24,29]. It is worth noting that seasonal peaks vary from region to region. This convincing evidence suggested that scarlet fever was more likely to spread in spring and winter than in other seasons, which was consistent with the findings of previous studies and the data of World Health Organization (WHO) [11,27].

The study results showed that children aged 3–9 years (age appropriate for kindergarten or primary school) were the susceptible group of scarlet fever in Zhejiang Province, accounting for 86.43% of the total cases. According to the WHO and the UK Public Health Department, scarlet fever infection is most common among children aged 5–15 [27,28]. Additionally, boys had a consistently higher incidence than girls, suggesting that boys were more prone to infection than girls. Similar results have been reported in some cities China as well as Poland [6,29,30]. Generally, boys participate in more group activities and care less about personal hygiene, which can increase the chances of pathogen exposure. Considering these findings, some conventional measures would be very effective in preventing and controlling scarlet fever. First, teachers and parents should instruct school-age children to wash their hands more often, which would be the best way to prevent this disease [31]. Second, schools should improve their environmental sanitation by disinfecting toys, handrails, desks, etc. [32]. In addition, more physical activities should be organized for children to enhance their health. Public health authorities should implement more effective surveillance for scarlet fever, and strengthen the disinfection efforts so that they may help reduce the risk and harm of scarlet fever epidemics to children.

From map of the incidence, the epidemic characteristics of scarlet fever showed the incidence in the northern regions was generally higher than that in the southern regions in 2004–2018 in Zhejiang Province. Shaoxing, Hangzhou, and Jiaxing were high-incidence regions, so the prevention and control effects of scarlet fever in these areas should be strengthened. Compared with the central and northern regions, the incidence was relatively low in other regions. The clusters of scarlet fever were displayed intuitively and comprehensively by geographic information system in Zhejiang Province. Getisd-ord Gi* statistics showed that high values clustered in the northern and central regions (Jiaxing, Huzhou, Shaoxing, Hangzhou, Jinhua), while rarely occurring in the eastern, western, and southern regions. The hotspot areas of scarlet fever showed a tendency to expand from the northern to the central regions since 2011. More high-value clusters had been detected in the north and central regions, because these areas maybe have similar topography, climate conditions, and other environmental factors which can easily lead to the spread of disease [22,24,25,33]. Further studies should be done to explore on the correlation between different latitudes and the incidence of scarlet fever [34]. These findings may help to guide scarlet fever control programs and targeting the intervention. By reason of information limitations, this study cannot fully consider other factors (such as drug resistance of strains, medical and health conditions, social and economic factors, etc.). Therefore, other key influencing factors of scarlet fever epidemic need to be further studied.

## 5. Limitations

Despite the above findings, our research had some limitations. First, as some mild cases may have used family therapies, and some cases of atypical symptoms may have been misdiagnosed, the reported data may underestimate the incidence. Second, case information was acquired from the CISDCP—a passive monitoring system—99.31% of cases were clinically diagnosed cases, and there is no corresponding laboratory testing. Therefore, we could not study the changes of circulating strains in 2004–2018, while this factor exactly played a significance role in explaining the rising incidence of scarlet fever in Zhejiang Province. Third, meteorological factors such as temperature, rainfall, and precipitation may affect scarlet fever epidemics, but we had no access to include such data in our study.

## 6. Conclusions

The epidemics of this disease showed obvious seasonal changes and geographic characteristics. Children under the age of 15 were more susceptible to infect scarlet fever, and children aged 3–9 were the main victims. Kindergartens and primary schools should be the focus of surveillance and control of this common disease. The scarlet fever epidemics mainly occurred in the north-central regions of Zhejiang Province.

## Figures and Tables

**Table 1:** Demographic characteristics of scarlet fever cases in Zhejiang Province, 2004–2018.

Group	2004	2005	2006	2007	2008	2009	2010	2011	2012	2013	2014	2015	2016	2017	2018	Total
Gender																
Male	331	406	602	551	530	407	278	1239	1092	714	1127	2159	1486	1391	1402	13,715
Female	193	244	328	322	286	242	177	704	614	503	726	1331	987	917	905	8479
Sex Ratio	1.72	1.66	1.84	1.71	1.85	1.68	1.57	1.76	1.78	1.42	1.55	1.62	1.51	1.52	1.55	1.62
Age																
0–2 years	35	35	64	51	72	54	68	136	95	85	126	194	158	106	131	1410
3–9 years	413	531	757	712	652	505	355	1692	1473	1036	1621	3117	2189	2072	2054	19,179
10–14 years	59	75	80	73	70	62	19	95	109	84	90	155	105	110	102	1288
≥15 years	17	9	29	37	22	28	13	20	29	12	16	24	21	20	20	317
Occupation																
Scattered children	120	153	171	174	245	164	157	384	315	254	355	636	500	473	413	4514
Kindergarten children	184	256	403	352	289	255	165	875	753	509	808	1650	1153	962	949	9563
Students	209	236	334	324	272	213	125	659	613	444	679	1187	806	864	929	7894
Others	11	5	22	23	10	17	8	25	25	10	11	17	14	9	16	223

(Scattered children are infants and young children who do not go to kindergarten. Kindergarten children usually refer to children 3–6 years old who spend their daytime at kindergarten. Others refer to all occupations except scattered kindergarten children and students.).

**Table 2:** Local spatial autocorrelation of scalet fever in Zhejiang Province in 2004–2018.

Year	Area	Gi Z-Score	Gi *p*-Value	Cluster
2004	No significant areas	-	-	-
2005	Huzhou	2.244472	0.024802	Hotspot
2005	Hangzhou	1.920238	0.054828	Hotspot
2006	Zhoushan	2.224427	0.02612	Hotspot
2007	Lishui	−1.736641	0.08245	Coldspot
2008	No significant areas	-	-	-
2009	Wenzhou	−2.105993	0.35205	Coldspot
2009	Lishui	−2.565872	0.10292	Coldspot
2009	Huzhou	1.870962	0.06135	Hotspot
2010	Jinhua	1.995008	0.046042	Hotspot
2011	Lishui	−1.664144	0.096084	Coldspot
2011	Hangzhou	1.881472	0.059908	Hotspot
2011	Shaoxing	2.012153	0.044204	Hotspot
2011	Jinhua	1.819235	0.068876	Hotspot
2012	Hangzhou	2.138269	0.032495	Hotspot
2012	Jiaxing	2.250799	0.024398	Hotspot
2012	Huzhou	1.967822	0.049088	Hotspot
2013	Shaoxing	2.2472	0.024627	Hotspot
2013	Jiaxing	2.107303	0.035091	Hotspot
2013	Huzhou	1.703672	0.088442	Hotspot
2014	Shaoxing	2.143372	0.032083	Hotspot
2014	Jiaxing	1.677282	0.093487	Hotspot
2014	Huzhou	1.910595	0.056057	Hotspot
2015	Shaoxing	2.455428	0.014072	Hotspot
2016	Shaoxing	2.181783	0.029126	Hotspot
2016	Jiaxing	1.657274	0.097464	Hotspot
2017	Shaoxing	2.083044	0.037247	Hotspot
2018	Shaoxing	1.929722	0.053641	Hotspot
2018	Jiaxing	1.775277	0.075852	Hotspot
